# Progress on quantum transport engineering in atomically precise anisotropic nanoporous graphene

**DOI:** 10.1039/d5na00532a

**Published:** 2025-08-26

**Authors:** Isaac Alcón, Aron W. Cummings, Esteve Ribas, Stephan Roche, Aitor Mugarza

**Affiliations:** a Institute of Theoretical and Computational Chemistry (IQTC), Department of Materials Science and Physical Chemistry, Universitat de Barcelona C/ de Martí i Franquès, 1-11, Les Corts 08028 Barcelona Spain ialcon@ub.edu; b Catalan Institute of Nanoscience and Nanotechnology (ICN2), CSIC and BIST Campus UAB, Bellaterra 08193 Barcelona Spain aitor.mugarza@icn2.cat; c ICREA, Institució Catalana de Recerca i Estudis Avançats 08070 Barcelona Spain

## Abstract

Bottom-up on-surface synthesis has demonstrated an impressive capability to realize desired carbon nanomaterials with atomic precision, also referred to as carbon nanoarchitectures. By using chemically tailored organic building blocks, it is possible to obtain virtually any carbon nanoarchitecture, with equally tunable electronic and magnetic properties. Among all known carbon nanoarchitectures, graphene nanoribbons (GNRs) have become the most extensively studied for nanoelectronics, due to their conductive π-conjugated structure and semiconducting nature. In this review, we summarize the progress made on a particular type of nanoporous graphenes (NPGs), conceived as 2D arrays of laterally bonded GNRs. Due to their relative novelty, these GNR-based NPGs have not yet acquired the same global attention as their predecessors (GNRs). However, recent progress suggests that these nanomaterials may play a central role in future carbon nanoelectronics and spintronics. This is due in large part to the ability to fine tune, both by chemical design and by external means, the electronic coupling between neighbouring GNRs within the NPG, thereby enabling precise control over the anisotropic properties of these materials, as demonstrated by various theoretical studies. In this review, we summarize the different approaches that have been proposed to tune such inter-ribbon coupling and, thus, the anisotropy. Overall, these studies underscore the unique platform that GNR-based NPGs provide for tailoring quantum electronic properties and two-dimensional anisotropy. As the field progresses, this capability could be harnessed for targeted applications at the molecular scale or even the atomic scale.

## Abstract 2

Here we review recent progress on atomically-precise anisotropic nanoporous graphenes (aNPGs) and their predicted highly tunable quantum transport anisotropy.

## Graphical abstract

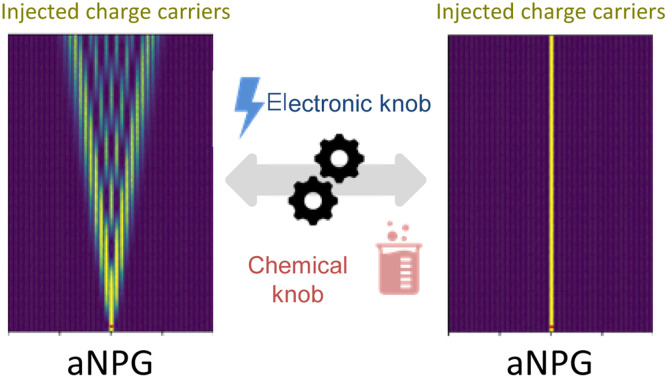

## Introduction

Bottom-up nanoarchitectonics, a chemical approach to synthesizing nanomaterials using specific molecular precursors, has demonstrated the capability to control nanomaterial structure with atomic precision.^1,2^ The surface-assisted synthesis of carbon-based one-dimensional nanostructures *à la carte* distinctly illustrates the power of this concept.^3,4^ This synthesis is based on transformative Lego chemistry, where molecular building blocks are self-assembled while internally transforming, giving rise to the desired nanoarchitecture. The resulting product depends on the rational design of the molecular precursor, as well as the controlled sequence of typically thermally activated reaction steps carried out on a metallic surface. Due to their fully π-conjugated and semiconducting nature, carbon nanoarchitectures have acquired significant attention as platforms for carbon nanoelectronics. The most prominent examples are bottom-up grown graphene nanoribbons (GNRs), which have been deeply studied for their use in solid-state devices.^4^ In particular, GNRs are currently being considered for beyond-silicon field-effect transistors, both as channel materials but also for interconnects.^5^ Additionally, the possibility to engineer, with atomic precision, exotic quantum phases of matter in these systems, such as non-trivial topological states or metallic spin chains, opens the door to exploit GNRs for quantum electronics with functionality beyond standard field-effect transistors.^6,7^

**Fig. 1:**
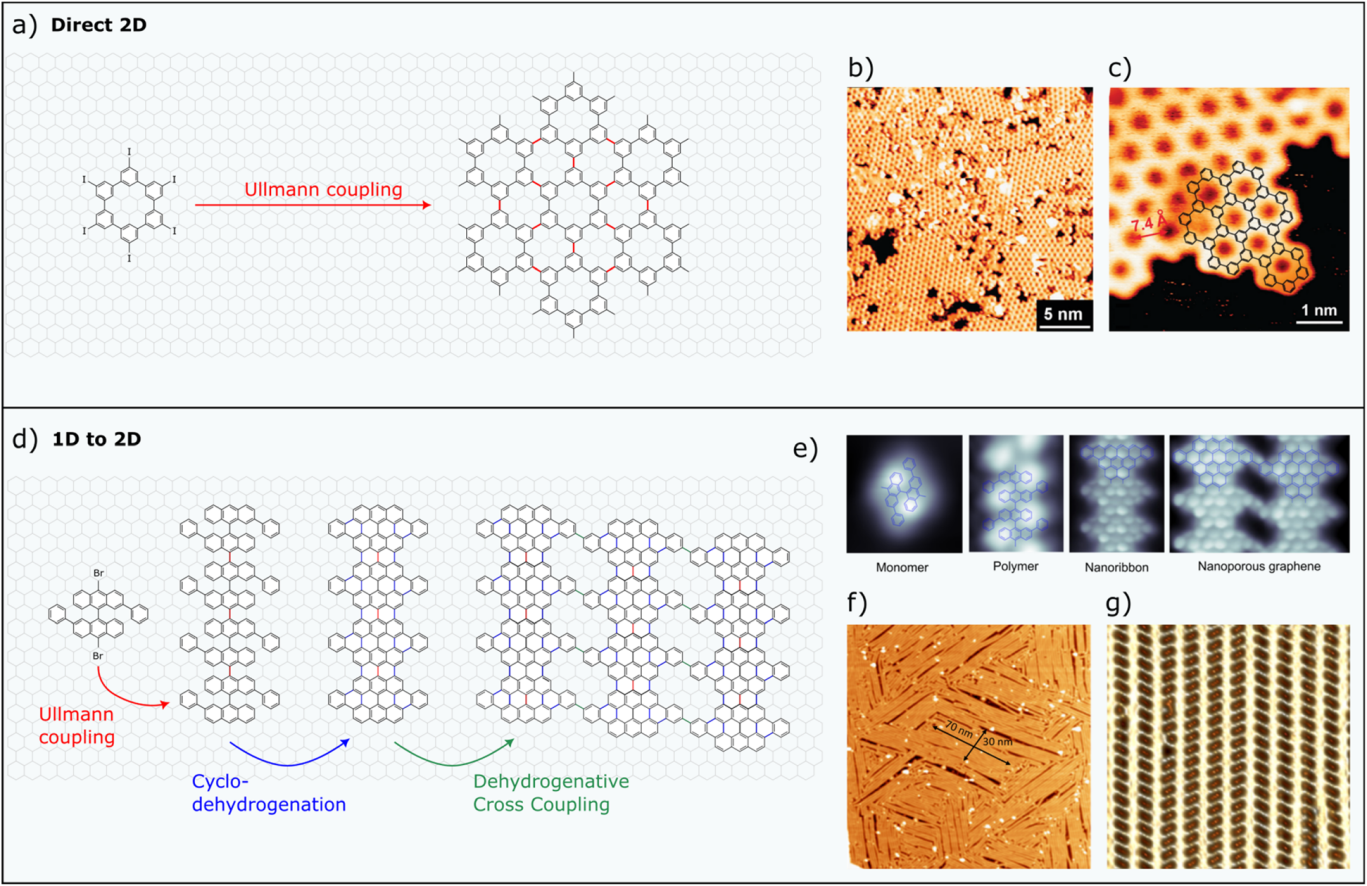
Different bottom-up strategies for the synthesis of 2D nanoporous networks. (a–c) Example of the first carbon-based porous network obtained by the direct 2D on-surface synthesis method. (a) Schematic illustration of the reaction. (b) STM image of the 2D network and (c) close up image with the chemical structure superimposed. (d and e) Example of the first carbon-based porous network obtained by the 1D to 2D on-surface synthesis method. (d) Schematic illustration of the sequential pathway. (e) Bond-resolved STM images of precursor, intermediaries and final product. (f) Large-scale STM image of the resulting polycrystalline aNPG membrane (size: 180 nm × 180 nm). (g) Close-up image of a single crystal domain (size: 18 nm × 18 nm). Images from (b) and (c) reproduced from ref. 10 with permission from the Royal Society of Chemistry, copyright 2009. Images from (e)–(g) reproduced from ref. 9 with permission from Science AAAS, copyright 2018.

More recently, growing attention has been directed toward a distinct class of on-surface synthesized carbon nanoarchitectures: the family of nanoporous graphenes (NPGs; see Fig. 1).^8^ Among the various types, those formed through the covalent coupling of GNRs stand out for their pronounced anisotropy.^9^ Their structural arrangement preserves the semiconducting behaviour of the individual GNRs, while introducing subtle perturbations from weak inter-ribbon electron coupling (Fig. 1d–g). A series of theoretical studies carried out in the last decade has shown that this inter-GNR coupling within NPGs, and the resulting degree of transport anisotropy, is highly tailorable *via* specific molecular design. This additional knob suggests that anisotropic NPGs, as the ones obtained from the coupling of GNRs, will become increasingly appealing for future applications, in particular for those related to carbon nanoelectronics, nanocircuitry, and nanosensing.

In this mini review we summarize the research being carried out on these GNR-based NPGs, hereafter referred to as anisotropic (a)NPGs, by introducing their synthesis and experimental characterization and later focusing on the theoretical studies assessing the quantum transport properties of these nanomaterials. Therefore, the topics we cover include: (1) the bottom-up synthesis of aNPGs and their atomically-resolved characterization, (2) the anisotropic transport properties of aNPGs, (3) how this anisotropy responds to different types of disorder, and (4) the use of aNPGs to induce anisotropic transport in other 2D materials *via* proximity effects. We aim to provide a picture of the state-of-the-art in aNPG fabrication, characterization and quantum transport modelling, with special focus on the tuneable transport anisotropy of these unique carbon nanoarchitectures.

## Bottom-up synthesis of anisotropic nanoporous graphene

Despite impressive advances in the bottom-up synthesis of GNRs, translating this method to two dimensional (2D) structures faces major challenges. Difficulties of extending covalent coupling reactions to 2D arise mainly from the fact that the creation of defects and domain boundaries is inherently more prevalent in 2D systems. This generally leads to defective, few nanometer scale domains^10–21^ – with a single exemption where domains, though still defective, could reach the 100 nanometer scale.^22^ The example of the first demonstration of an on-surface synthesized 2D carbon-based covalent network, obtained using a direct 2D synthesis method, is displayed in Fig. 1a–c.

The challenges inherent in direct 2D covalent synthesis can be addressed through a sequential strategy in which one-dimensional (1D) units are first synthesized and subsequently laterally coupled to form a 2D porous network. This approach leverages the significantly lower defect rate associated with 1D synthesis and builds on over 15 years of successful graphene nanoribbon (GNR) fabrication. The porous interface also leads to highly anisotropic structures, where differences in π-electron delocalization along and across the ribbons can be substantial. When the underlying herringbone reconstruction of Au(111) serves as a template to align the 1D units, large-scale domains can form, with their azimuthal orientation constrained by the threefold symmetry of the surface reconstruction. In the pioneering study illustrated in Fig. 1d–g large anisotropic domains of up to 30 nm × 70 nm were shown to fuse *via* covalent bonding, producing macroscopic polycrystalline sheets characterized by three discrete domain orientations. The concept of fusing GNRs or 1D polymers to construct 2D porous networks has since been adopted in several follow-up studies.^23–25^ One of these studies demonstrated that, when the connecting units exhibit localized states near the Fermi level, the lateral fusion of GNRs can give rise to additional frontier electronic bands localized at the connectors, coexisting with the GNR conduction and valence bands.^23^

The combination of the sequential 1D-to-2D synthetic strategy with edge functional groups that act as molecular connectors enables the design of a diverse portfolio of aNPG structures sharing the same backbone GNRs but with different inter-ribbon coupling, that is, different electronic interaction between neighbouring GNRs within the NPG crystal. This concept of molecular bridge engineering was exemplified by modifying the original molecular precursor from ref. 9 through the addition of phenyl substituents, resulting in the incorporation of bisphenyl (BPh) units between adjacent 7-13-AGNRs to form BPh-aNPG.^26^ In this structure, the multiplicity of inter-ribbon coupling results in *meta*–*meta* (*mm*), *meta*–*para* (*mp*), and *para*–*para* (*pp*) bridge configurations, similar to the *mm* and *pp* bridges previously observed at the local scale in coupled Chevron GNR pairs.^27^

However, unlike direct 2D synthesis, where covalent networks can form in a single reaction step at relatively mild temperatures, the sequential 1D-to-2D approach involves hierarchical steps of increasing temperatures, challenging the stability of the heteroatoms and functional groups. This poses challenges to their thermal stability, potentially leading to functional group transformations^23,28^ or inter-ribbon reactions.^29^

To mitigate this, one can minimize reaction temperatures and avoid unwanted inter-ribbon reactivity. A solution for the latter is the realization of GNR arrays that define nanoscale channels that act as confined reaction environments—nanochannel reactors—where a second, interdigitated molecular component can be synthesized in isolation. In a subsequent step, this second component can be covalently coupled to the adjacent “templating” GNRs. If the two GNR components have different electronic structure, an aNPG with a built-in heterostructure superlattice is formed, as recently demonstrated.^30^ In this case, the inter-ribbon coupling could be electrostatically controlled by modulating the band alignment between the two components.

## Quantum interference and anisotropic properties

**Fig. 2:**
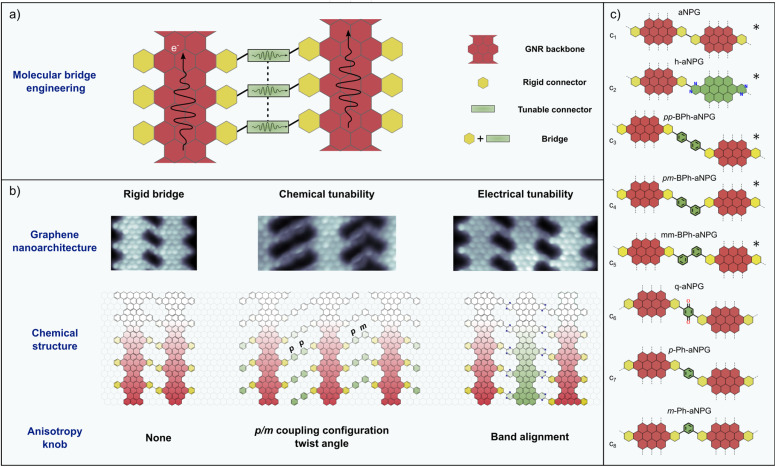
Molecular bridge engineering concept. (a) Schematic illustration of the modular nature of the anisotropic nanoporous graphene structures that are the focus of this review. (b) Bond-resolved STM images of three aNPG structures obtained by on-surface synthesis. The left one is directly coupled through the connector benzenes,^9^ the center one has bisphenyl bridges with different coupling configurations that can be seen as modulators of the inter-ribbon coupling;^26^ the right one has interdigitated N-doped 7-13-AGNRs that can be conceived as bridges that couple the undoped GNRs.^30^ Alternatively, the whole nanostructure can be considered as a heterostructure superlattice. (c) Summary of bridge configurations that have been reported, labelled from C_1_ to C_8_ and with the corresponding reference. The experimentally synthesized ones are marked with *. c_1_: ref. 9; c_2_: ref. 30; c_3_–c_5_: ref. 26; c_6_: ref. 39; c_7_, c_8_: ref. 36.

The experimentally demonstrated aNPGs described in the previous section, summarized in Fig. 2b (c_1_ to c_5_ bridge configurations in Fig. 2c), are characterized by a strongly anisotropic structure that is imprinted in all physical properties of the layers.

*Ab initio* calculations of the pioneering work demonstrated that bottom-up-synthesized aNPG is a material with coexisting longitudinal (L) and transversal (T) quasi-1D electronic bands.^9^ The absence of T bands in the −1 to +1 eV window around the Fermi level defines a region with very high electronic anisotropy, with a L/T conductance ratio of around 10–20. The synthesis of this aNPG inspired several further theoretical studies, revealing a high intrinsic mobility of 800 cm^2^ V^−1^ s^−1^ in the longitudinal direction, larger than that of MoS_2_ (400 cm^2^ V^−1^ s^−1^)^31^ or Si thin films (250 cm^2^ V^−1^ s^−1^),^32^ and a L/T ratio of around 4.^33^ Comparable anisotropies in the tensile strength and thermal conductivity were found in a different study.^34^

**Fig. 3:**
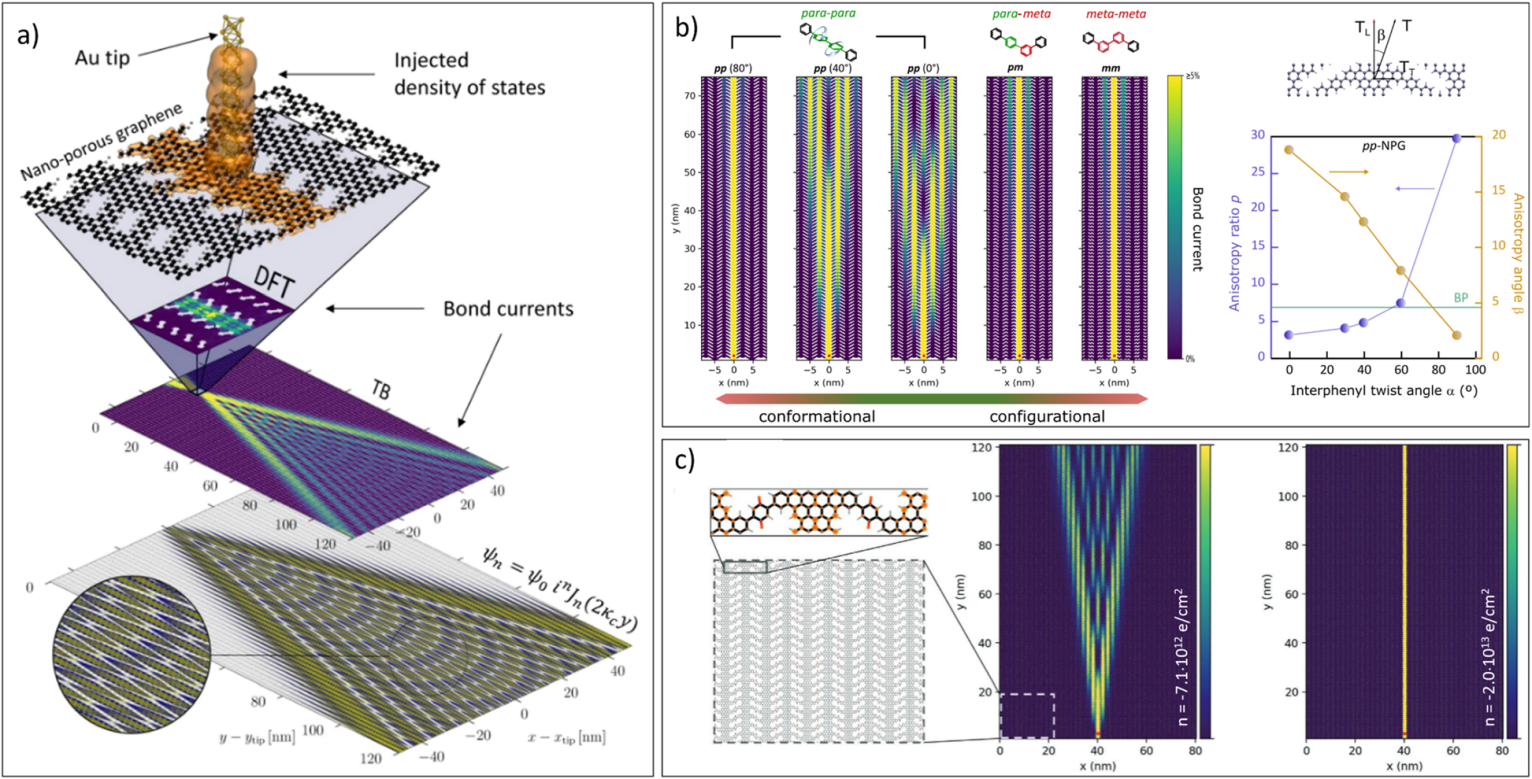
Talbot interference in aNPGs. (a) Details of the approach used to simulate quantum interference in the electron propagation within the aNPG obtained with the 7-13-AGNRs, as reported in ref. 9. Reproduced from ref. 35 with permission from ACS, copyright 2018. (b) Left: Modulation of Talbot interference by changing the *para*/*meta* bonding configuration within the bisphenyl bridge, and the twist angle in the case of the *pp*-BPh-aNPG. Right: Corresponding modulation of the anisotropy in the electron transmission as a function of the interphenyl twist angle in *pp*-BPh-aNPG. The green line represents the anisotropy of black phosphorous. Reproduced from ref. 26 with permission from ACS, copyright 2023. (c) Electrostatic modulation of Talbot interferences in q-aNPG. Reproduced from ref. 39 with permission from Wiley, copyright 2021.

The weakly coupled electron channels can also give rise to quantum interference (QI) phenomena, the electron analogue of the optical Talbot effect. This was studied by simulating the propagation along the aNPG of an electron injected in one of the ribbons (see Fig. 3a).^35^ The interference can be observed in a wide energy window of approximately ±0.7 eV around the Fermi level, where longitudinal transport channels are predominant.

This seminal theoretical work prompted several follow-up studies that considered structures beyond those synthesized to date, altogether summarized and labelled from c_1_ to c_8_ in Fig. 2c. The first such study considered the *para* and *meta* configuration of single phenyl bridges (*p*/*m*-Ph-aNPG) as a way of controlling the inter-ribbon coupling by means of constructive or destructive QI, respectively (c_7_ and c_8_).^36^ Simulations revealed that destructive QI could maintain the propagation of electrons along single GNRs for a distance of at least 100 nm. A similar conclusion was obtained for simulations of the experimentally realized BPh-aNPG (c_3_ to c_5_ in Fig. 2c). As shown in Fig. 3b, despite the inter-ribbon coupling being weaker due to the larger bisphenyl bridge, the *para–para* coupling still led to a Talbot-like spread of the propagating electron wave, whereas a single *meta* bond was enough to quench the electronic coupling and lead to an effective confinement within the GNR where the electron was injected.^26^

The presence of “leaky” *para*-phenyl bridges offers a different, more tunable knob to modulate the inter-ribbon coupling, which is the control of the π–π overlap by means of the inter-phenyl twist angle (see Fig. 3b). This concept was explored for BPh-aNPG, demonstrating that the maximum transmission found for coplanar bridges could be effectively quenched to negligible values by twisting the bridges to orthogonal inter-phenyl angles.^26^ In this way, the anisotropy could be tuned by one order of magnitude, reaching values 4 times larger than that of black phosphorous, a model anisotropic 2D material (see right panel in Fig. 3b). We anticipate that the bridge twist angle could be externally controlled by electric fields when using functionalized polar bridges, as theoretically proposed for molecular systems.^37,38^

Electrostatic gating seems to be a more obvious way of modulating QI in the bridges, if we assume that inter-ribbon coupling depends on the Fermi level position. Unfortunately, this does not occur in any of the aforementioned structures. The electron–hole symmetry and the rather featureless energy-dependent inter-ribbon transmission within the energy window of pure L bands results in a small electrostatic tunability. This, however, can be counteracted by introducing bridges with molecular frontier orbitals (HOMO and LUMO) close to the Fermi level, breaking the e–h symmetry and inducing energy-dependent inter-ribbon coupling. This is the case in the theoretically studied aNPG bearing *para*-benzoquinone bridge units (q-aNPG, c_6_ in Fig. 2c).^39^ The effective gate-dependent modulation of Talbot interference in this system can be clearly seen in Fig. 3c. One could further modulate the inter-ribbon coupling in a more robust fashion by considering the electrochemical reduction of the quinone oxygen groups with hydrogen atoms, as also shown in ref. 39.

The presence of molecular bridge states near the Fermi level enables the use of electrostatic gating to modulate their occupation. In the case of q-aNPG, this can be achieved through moderate n-type doping.^39^ Specifically, when quinoidal bridges are employed, charging the bridge introduces an open-shell character, leading to local spin polarization within every bridge unit. This spin-polarized state at the bridge gives rise to a spin-selective Talbot interference effect. Thus, at certain gating levels, one spin component of injected electrons becomes fully confined within the electrically probed GNR, while the other delocalizes and forms interference patterns. This Talbot spin-filtering mechanism may pave the way for novel spintronic functionalities in all-carbon nanocircuit architectures.

A recent study explores the use of a transversal electric field in the first reported aNPG formed with 7-13-GNRs (c_1_ in Fig. 2c).^40^ The transversal field-dependent evolution of the transmission maps shows a clear evolution from the Talbot interference patterns to oscillating patterns that represent the electronic analogue of the optical Bloch oscillations. The effect of increasing the electric field across the ribbons is to refocus the electron path with decreasing period and lateral extent. This electrically controlled quantum transport provides a flexible route to spatially configurable graphene-based nano-circuitry that can be applied in nanoelectronics, molecular sensing, or quantum information processing.

Finally, the transport properties of h-aNPG (c_2_ bridge) were recently theoretically evaluated. The authors found that c_2_ also displays a Talbot-like interference pattern, however, the spreading of currents is GNR-selective. That means that if currents are injected in a non-doped (doped) GNR, the resulting spreading of currents only takes place through the non-doped (doped) GNR array. This effect was found to originate from the band-staggering naturally embedded in the lateral heterostructure present in h-aNPG. Additionally, the authors explored different design strategies to confine injected currents in single-GNR channels.^41^ The most effective means to do so turned out to be the use of meta-configured phenyl bridges which, upon being combined with the band-staggering of the system, leads to ultra-long current confinement reaching the micron scale. Therefore, the combination of destructive QI engineering and band staggering could be the most effective strategy to apply aNPGs for carbon nanocircuitry applications, where electric signals could be sent with sub-nanometer precision for very long distances.

## Effect of disorder on transport anisotropy

Disorder plays an important role in any electronic device, but particularly in those devices based on low-dimensional systems such as 2D materials, leading to strong charge carrier scattering.^42–44^ For example, the effect of different types of disorder on charge transport has been studied numerically in graphene, including vacancies,^45^ impurities,^46^ chemical adsorbates^47,48^ and substrate effects.^49–51^ Dynamic disorder, mainly arising from thermal vibrations at finite temperature has also been studied.^51,52^ As previously explained, bottom-up on-surface synthesis provides a route to achieve atomic precision in carbon nanomaterials, though major challenges still exist towards that goal. Therefore, in the long run, one could expect atomically perfect carbon nanostructured devices to become a reality, thus avoiding major sources of charge-carrier scattering such as vacancies, structural dislocations or chemical impurities. However, even in such a futuristic scenario, carbon nanodevices would be subject to other important sources of disorder arising from the interaction with underlying substrates (these not necessarily being “atomically perfect”) or from thermal fluctuations at finite temperature. The effect of such “unavoidable” types of disorder on quantum transport in aNPGs has already been evaluated in two separate studies, as explained here.

Electrostatic disorder is the main form of disorder arising from the interaction of 2D materials with underlying substrates or from chemical adsorbates, the latter normally present in the device fabrication process.^49,53,54^ In 2023, the effect of electrostatic disorder on the quantum transport properties of *p*/*m*-Ph-aNPG, the *para*-configured single phenyl bridged NPG (*p*-Ph-aNPG), and *meta*-configured single phenyl bridged NPG (*m*-Ph-aNPG) was studied theoretically (c_7_ and c_8_ in Fig. 2c).^55^ The work, based on effective tight-binding models and linear-scaling quantum transport (LSQT) simulations,^56,57^ reached two major conclusions. The first was that the impact of electrostatic disorder on transport along GNRs is proportional to the NPG transport anisotropy. In other words, the more confined charge carriers are within GNRs, the more severe the effect of charge disorder is on quantum transport, due to the higher probability of back scattering.^55^

**Fig. 4:**
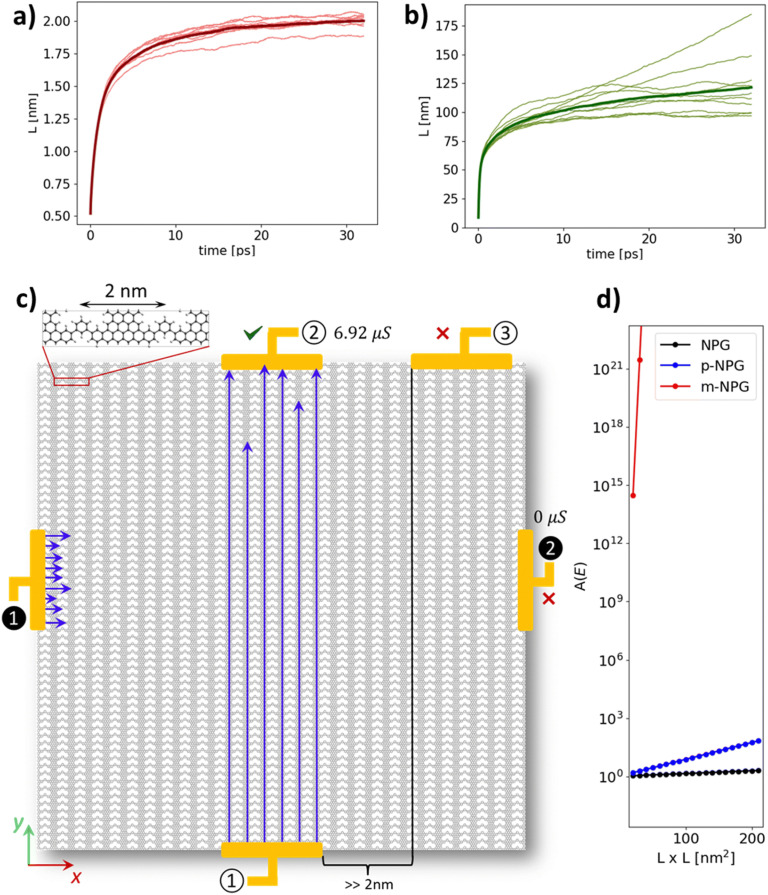
Wave packet propagation lengths (a) across GNRs and (b) along GNRs as computed with LSQT for m-Ph-aNPG (c_8_ in Fig. 2c). (c) Schematic representing the implications of results in (a) and (b) for different electrode setups measuring conductance along GNRs (① → ②) or across GNRs (① →③ and ❶ → ❷). (d) Comparison between quantum transport anisotropy (*A*) defined as the ratio of conductance along and across GNRs (*A*(*L*) = *G*_*y*_(*L*)/*G*_*x*_(*L*)) both calculated from the corresponding localization lengths. Reproduced from ref. 55 with permission from the Royal Society of Chemistry, copyright 2024.

The second key finding in this work was the demonstration of infinite anisotropy (*A* = ∞) in *m*-Ph-aNPG. This was demonstrated by estimating the length-dependent conductance (*G*_*x*_(*L*) and *G*_*y*_(*L*)) from the so-called localization length *L* calculated for each aNPG and transport direction (*i.e.* along and across GNRs), as shown in Fig. 4a and b for *m*-Ph-aNPG. Fig. 4d shows how anisotropy, defined as the ratio of conductance values along each direction, *A*(*L*) = *G*_*y*_(*L*)/*G*_*x*_(*L*), varies for different device lengths (*L*) for the different aNPGs. One can see the effective explosion of quantum transport anisotropy (*A*) in *m*-Ph-aNPG for any device larger than 20 × 20 nm^2^, far beyond the anisotropy of the other aNPGs (Fig. 4d). In effect, this means that a null conductance should be expected in *m*-Ph-aNPG devices for lengths larger than 5 nm in the perpendicular direction to GNRs (*e.g.* ❶ → ❷ in Fig. 4c), while simultaneously measuring finite conductance values in the parallel direction for lengths well beyond 100 nm (*e.g.* ① → ② in Fig. 4c).

**Fig. 5:**
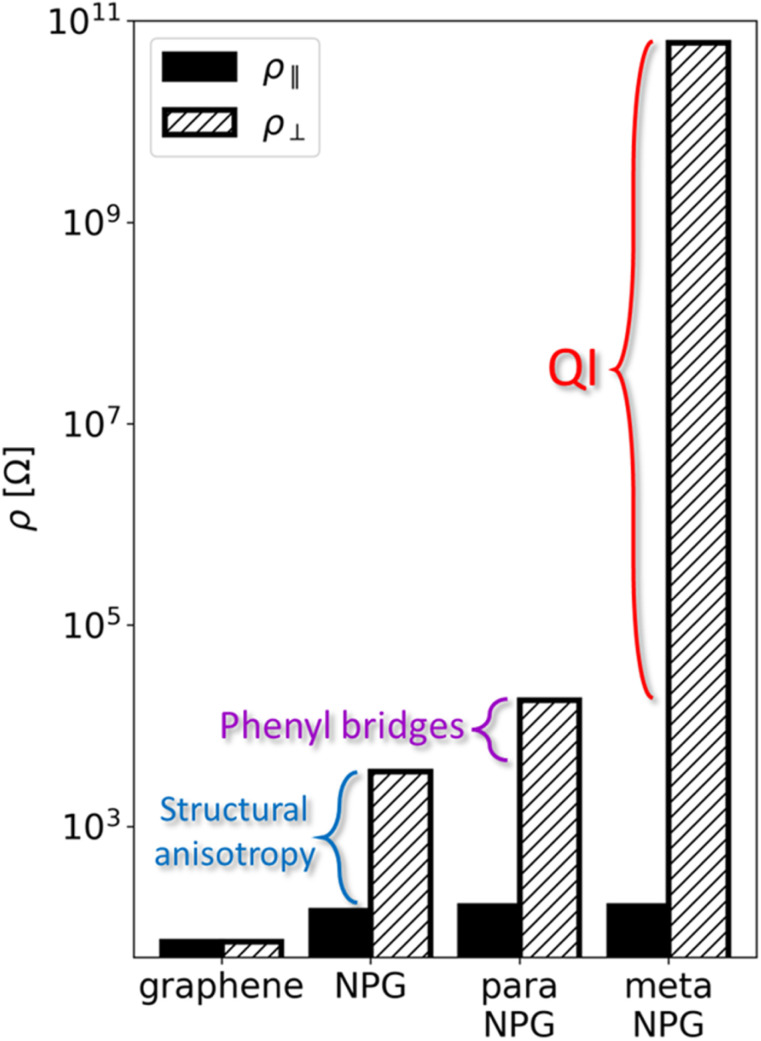
Comparison between calculated resistivity values along GNRs (*ρ*_∥_, filled bars) and across GNRs (*ρ*_⊥_, hatched bars) for the different aNPGs thermally activated at 300 K. The plot, in logarithmic scale, allows to distinguish the different structural and chemical parameters contributing to transport anisotropy in each case, with the chemical engineering (in this work referred to as quantum interference, QI, engineering) in *m*-Ph-aNPG emerging as the most effective means to induce giant anisotropy. We note that in this work *m*-Ph-aNPG was referred to as *meta*NPG, whereas *p*-Ph-aNPG was labelled as *para*NPG. Reprinted from ref. 58, Copyright (2025), with permission from Elsevier.

More recently, another contribution has analysed the role of dynamic disorder, that is thermal fluctuations, at 300 K.^58^ This work simulated the quantum transport properties of the same three aNPGs, that is the foundational aNPG (c_1_), *p*-Ph-aNPG (c_7_) and *m*-Ph-aNPG (c_8_), but using the nonequilibrium Green's function method.^59,60^ More specifically, this work used the so-called molecular-dynamics Landauer approach (MD-Landauer).^61^ This consisted of using MD simulations to thermally activate 100 × 100 nm^2^ aNPG samples (*ca.* 450 000 atoms) and calculating the electronic transmission through a series of extracted snapshots during the MD to obtain statistically averaged resistivity values along both in-plane directions (*ρ*_∥_ and *ρ*_⊥_, respectively). This work also reached two important conclusions. The first was that all considered NPGs display approximately the same resistivity along the GNRs. This contrasts with results obtained under electrostatic disorder, as previously explained.^55^ Therefore, varying the electronic coupling between GNRs does not significantly influence their transport capability under dynamic disorder at 300 K. The second key finding, like the previous work,^55^ was that the effectiveness of *meta*-configured phenyl bridges to cut transport across GNRs remained fully operational under thermal fluctuations and even improved upon the 0 K scenario. At a device length of 25 nm, electronic transmission in the direction across the GNRs was computed to be of the order of 10^−8^ or, effectively, zero transmission. With this, the authors demonstrated that *meta*-connections between GNRs (referred to as QI engineering in that work) are by far the most effective means to induce giant quantum transport anisotropy in aNPG systems at finite temperature, as depicted by the comparison of resistivity values along GNRs (*ρ*_∥_) and across GNRs (*ρ*_⊥_) shown in Fig. 5 for all considered aNPGs, plus graphene as a ref. 58.

## Transport anisotropy *via* proximity effects

An additional key feature of NPGs is that, like graphene and other 2D materials, they are all surface, and thus they may be used to modify the properties of other 2D materials by stacking them together.^62^ Generally, such proximity effects may be used to combine the ideal properties of one 2D material with those of another. For example, it is possible to greatly enhance the spin–orbit coupling (SOC) in graphene by layering it with large-SOC materials such as transition metal dichalcogenides or topological insulators.^63,64^ Such heterostructures maintain graphene's Dirac-like band structure and superior charge transport properties while also imbuing it with unique spin textures and spin transport properties^65–67^ that may be utilized in spintronics applications.^68,69^

**Fig. 6:**
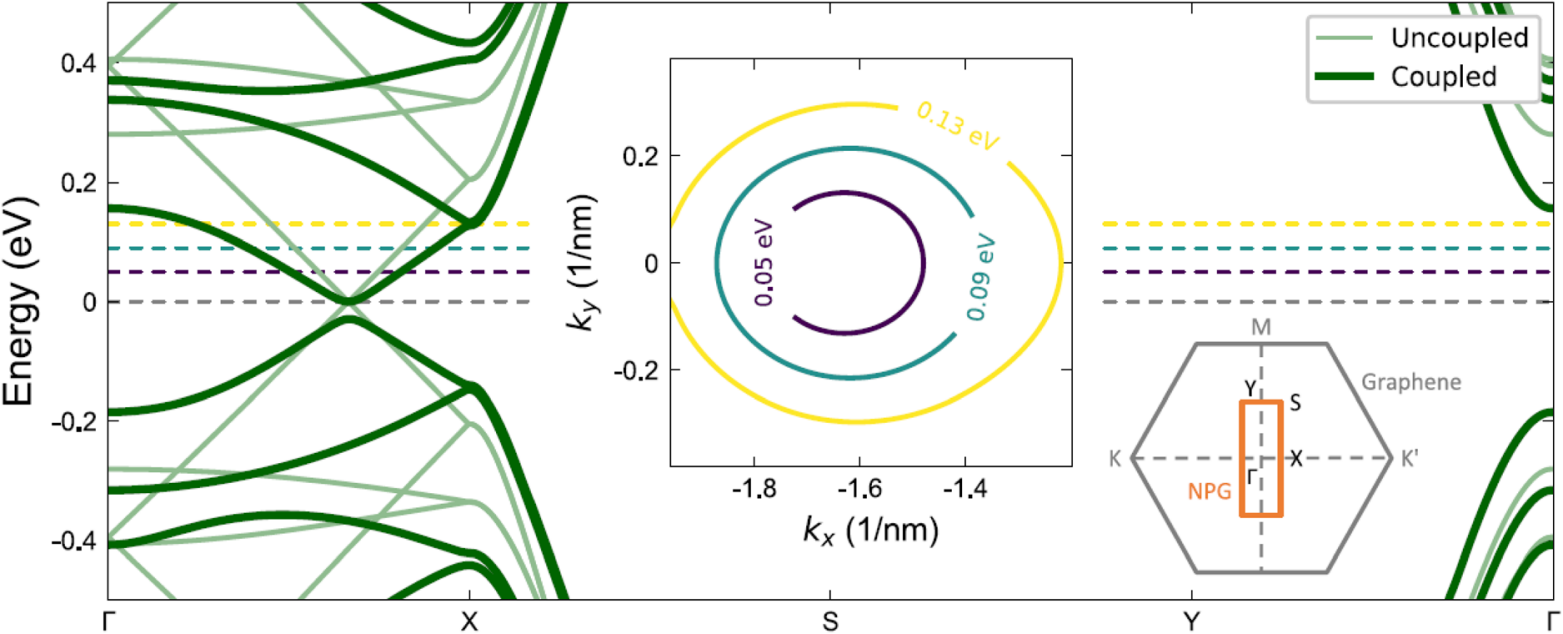
Band structure of the Bernal-stacked graphene/aNPG heterostructure. The dark (light) lines show the coupled (uncoupled) system. The middle inset shows the Fermi surface of the graphene bands at three different energies, indicated by the dashed horizontal lines in the main panel. The oval shape reflects anisotropy induced by the NPG layer. Reproduced from ref. 70 under the terms of the Creative Commons CC BY license.

In a similar vein, a few studies have examined the possibility of altering the band structure of graphene by layering it with aNPGs (c_1_).^70–72^ The first such study we are aware of looked at this in the context of optical applications, with the goal of inducing optical anisotropy in graphene while preserving its Dirac bands and broadband optical absorption.^70^ It was found that in graphene/NPG bilayer heterostructures, the graphene bands come anisotropic, as shown in the middle inset of Fig. 6, with ratio of Fermi velocities along the *y*- and *x*-directions, *v*_*y*_/*v*_*x*_ = 1.2–1.4, depending on the Fermi level. Similar ratios were also found in the charge transport anisotropy *σ*_*yy*_/*σ*_*xx*_, where *σ*_*jj*_ is the charge conductivity along the *j* direction, and in the optical anisotropy *σ*_*y*_(*ω*)/*σ*_*x*_(*ω*), where *x*/*y* denotes the polarization of the incoming light and *ω* its frequency.

This work also studied the effect of different types of NPGs (the foundational aNPG, *p*-Ph-aNPG, *m*-Ph-aNPG) as well as arrays of GNRs. It found that the anisotropy induced in the graphene layer scales inversely with the bandgap of each aNPG material, with the highest anisotropy induced by aNPG and the lowest by *m*-Ph-aNPG. The same trend was found for arrays of GNRs on graphene: that is, by lowering the band gap of the material interfaced with graphene (*i.e.* by using wider ribbons), one could increase the anisotropy ratio to 1.6 in the graphene layer. For band gaps below this critical value, the anisotropy was then found to decrease again, indicating the presence of an optimal ribbon width for inducing charge transport anisotropy in graphene.

A subsequent work examined graphene/aNPG heterostructures with the goal of tuning the band gap in the graphene layer *via* a perpendicular electric field.^71^ They found two different mechanisms of gap opening: (1) symmetry breaking in Bernal-stacked graphene/aNPG, with a band gap linearly proportional to the applied electric field; or (2) merging of Dirac points in AA-stacked graphene/aNPG, with a band gap that obeyed a diode-like behavior, only opening after reaching a critical electric field. Their models were supported by DFT calculations indicating that band gaps on the order of 100 meV could be reached for reasonable electric field values.

**Fig. 7:**
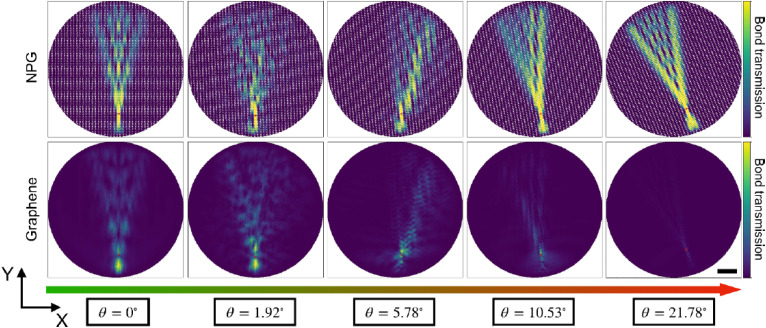
Charge transport in twisted graphene/NPG with current injected in the NPG layer. The top (bottom) row shows carrier transport in the NPG (graphene) layer, indicating decoupling of the layers at large twist angle. The scale bar is 10 nm. Reprinted with permission from ref. 72. Copyright (2025), American Chemical Society.

In the context of the recent interest in the field of twistronics, which aims to tune the properties of 2D material multi-layer structures *via* their twist angle,^73^ a recent work examined the role that twist angle between graphene and aNPG has on the charge transport properties.^72^ Here the goal was to find a suitable substrate that could preserve the anisotropic transport properties of isolated aNPGs. It was found that at low twist angles, graphene and aNPG were strongly coupled, with anisotropic transport induced in the graphene layer as well as chiral transport in both layers owing to in-plane symmetry breaking. Meanwhile, at large twist angles, the two layers were effectively decoupled, and the transport properties of isolated aNPG and graphene were restored. These transport results are summarized in Fig. 7.

## Conclusions

Since the seminal work demonstrating bottom-up synthesis of anisotropic nanoporous graphene (aNPG),^9^ significant progress has been made both in the fabrication of novel aNPGs and in the theoretical unveiling of their unique transport characteristics. This ensemble of efforts has shown that the physicochemical properties of aNPGs are highly tunable *via* rational molecular design. Different studies have demonstrated that the inter-GNR coupling may be finely tuned by either (i) molecular-bridge engineering (“chemical” means in Fig. 2) or (ii) shifting the chemical potential of neighboring GNRs (“electronic” means in Fig. 2). Both structures have been realized experimentally, and accurate quantum transport simulations have shown the profound effect of such chemical and electronic knobs to tune the quantum transport anisotropy of the system.

Among the different mechanisms, *meta*- and *para*-configurations of phenyl and biphenyl bridges appear, to date, as the most effective means to control inter-ribbon coupling and thus transport anisotropy. *Meta* connections have been shown to effectively quench quantum transport across GNRs.^26,36^ On the other hand, *para* connections allow a more gradual tuning of inter-ribbon coupling *via* a phenyl out-of-plane rotation.^26^ The robustness of these mechanisms to tune quantum transport has also been evaluated in the presence of electrostatic and dynamic disorder. The main conclusion of these works is that transport anisotropy in aNPGs survives disorder while, in turn, impacting the inherent conductivity along GNR channels. The discovery of giant quantum transport anisotropy is another notable finding from this latest research.^55,58^ Finally, aNPGs may form van der Waals heterostructures with other 2D materials to imprint their anisotropic behaviour. This has been shown in theoretical studies, with optical and charge conductivity in graphene becoming anisotropic due to interaction with aNPGs.^70,72^ The degree with which such anisotropy is induced in graphene depends on its effective coupling with the aNPG layer. This inter-layer coupling, in turn, may be tuned *via* (i) the aNPG band gap,^70^ (ii) a perpendicular electric field,^71^ or (iii) *via* twisting the aNPG layer with respect to graphene.^72^

The plethora of studies summarized here highlights the richness that these novel carbon nanoarchitectures, aNPGs, may offer in the future for advanced technological applications and, concretely, for carbon nanoelectronics and spintronics. Despite the variety of results already reported, the number of aNPG structures is still limited, especially if compared to the chemical and structural diversity offered by synthetic organic chemistry. Furthermore, experimental access to the quantum anisotropy of these materials has so far been constrained by the difficulty of fabricating or precisely positioning deterministic electrodes at the nanometer scale. Therefore, the significant advancements made to date may only be the tip of the iceberg, which we anticipate will keep revealing itself in the coming years or even decades. Future progress in on-surface synthesis, device integration and theoretical modelling will promote the acceleration of aNPG research on all fronts and, as of today, it is hard to see the ceiling for such a versatile, fascinating, and novel class of carbon nanomaterials.

## Author contributions

Isaac Alcón: conceptualization, writing – original draft, writing – review & editing. Aron W. Cummings: conceptualization, writing – original draft, writing – review & editing. Esteve Ribas: writing – review & editing. Stephan Roche: conceptualization. Aitor Mugarza: conceptualization, writing – original draft, writing – review & editing.

## Conflicts of interest

The authors have no conflicts of interest to declare.

## Data Availability

No primary research results, software or code have been included and no new data were generated or analysed as part of this review.
